# Psychometric properties of the EQ-5D-5L compared with EQ-5D-3L in cancer patients in Iran

**DOI:** 10.3389/fonc.2022.1052155

**Published:** 2022-12-09

**Authors:** Nasrin Moradi, Thomas G. Poder, Hossein Safari, Mohammad M. Mojahedian, Hosein Ameri

**Affiliations:** ^1^ Department of Health Management and Economics, School of Public Health, Iran University of Medical Science, Tehran, Iran; ^2^ Department of Management, Evaluation and Health Policy, School of Public Health, University of Montreal, Montreal, QC, Canada; ^3^ Centre de recherche de l’Institut universitaire en santé mentale de Montréal, CIUSSS de l’Est de l’île de Montréal, Montreal, QC, Canada; ^4^ Health Promotion Research Centre, Iran University of Medical Sciences, Tehran, Iran; ^5^ Department of Pharmacoeconomics, School of Pharmacy, Iran University of Medical Science, Tehran, Iran; ^6^ Health Policy and Management Research Center, Department of Health Management and Economics, School of Public Health, Shahid Sadoughi University of Medical Sciences, Yazd, Iran

**Keywords:** EQ-5D-5L, EQ-5D-3L, psychometric properties, cancer patients, Iran

## Abstract

**Background and Objective:**

Psychometric evidence to support the validity and reliability of the EuroQol-5 Dimensions (EQ-5D) in cancer patients is limited. This study aimed to test the validity and reliability of the EQ-5D-5L (5L) in comparison with EQ-5D-3L (3L) in cancer patients.

**Methods:**

Data of 650 cancer patients were collected through consecutive sampling method from three largest governmental cancer centers in Iran between June 2021 and January 2022. The data were gathered using the 3L, 5L, and the European Organization for Research and Treatment of Cancer quality of life questionnaire (QLQ-C30) instruments. The 3L and 5L were compared in terms of ceiling effect, discriminatory power, convergent and known-groups validity, relative efficiency, inconsistency, agreement, and reliability.

**Results:**

Compared with the 3L, ceiling effect decreased by 27.86%. Absolute and relative informativity of discriminatory power improved by 45.93% and 22.92% in the 5L, respectively. All convergent validity coefficients with 5L were stronger than with 3L. Both 3L and 5L demonstrated good known-groups validity, and the relative efficiency was higher for 5L in 4 out of 7 patients’ characteristics. The two instruments showed low overall inconsistency (1.45%) and 92.57% of the differences of observations between the 3L and 5L were within the 95% limit of agreement. The interclass correlation coefficient (ICC) for 3L and 5L indexes were 0.88 and 0.85, respectively, and kappa coefficients in the 3L dimensions (range=0.66-0.92) were higher than the 5L(range=0.64-0.79).

**Conclusions:**

The 5L demonstrated to be better than the 3L in terms of ceiling effect, inconsistency, discriminatory power, convergent validity, relative efficiency.

## Introduction

Cost-utility analysis (CUA) is one of the economic evaluation approaches which is widely used for assessing healthcare interventions (1). CUA evaluates interventions in terms of cost per quality adjusted life-years (QALYs) gained, where QALYs combine length of life and health-related quality of life (HRQoL) into a single index ranging from 0 (death) to 1 (perfect health), with negative values indicating health states worse than death (2). HRQoL is a multidimensional construct widely used to assess the impact of health status on quality of life (3). In order to calculate QALYs, preference-based HRQoL instruments such as the EuroQol-5 Dimensions (EQ-5D) and the Short-Form 6 dimensions (SF-6D) are widely used (4). The EQ-5D is the most popular type of preference-based instrument that is developed based on general dimensions of health, and can be used in both clinical trials and health services research (5).

EQ-5D is commonly used in versions: EQ-5D-3L (3L), EQ-5D-5L (5L), and Youth’ versions of the EQ-5D (EQ-5D-Y-3L and EQ-5D-Y-5L). The 3L consists of two parts: a classification system of five dimensions (mobility, self-care, usual activities, pain/discomfort, and anxiety/depression) with 3 levels of response options (no problems, some or moderate problems, and extreme problems) per each dimension and a visual analogue scale (VAS). The classification system of 3L generates 243 (i.e., 3^5^) possible health states. The VAS records the current self-rated health of respondents on a vertical line ranging from 0 (the worst imaginable health) to 100 (the best imaginable health). The new version of EQ-5D, the 5L includes a classification system of five dimensions with 5 response levels per each dimension (no problems, slight problems, moderate problems, severe problems, and unable to/extreme problems) and a VAS. The 5L classification system defines 3125 (i.e., 5^5^) possible health states. The EQ-5D-Y is used for adolescents between 7 to 12 years, which includes 5 dimensions with 3 levels and is labelled in a way that children can easily understand it (6).

Increasing evidence showed that the 5L has gained a widespread attention in studies, because expanding the range of responses from three to five levels on each of the dimensions has increased the instrument’s sensitivity and decreased its ceiling effect threshold (7). This version has been translated into more than 100 languages including Persian, and its psychometric properties have been assessed in comparison with the 3L in various general and patient groups (8–11), but not in a developing country.

Given the growing number of cancers and available treatments, an increasing use of the 5L to perform CUA and to assess the HRQoL in cancer patients is expected in the years to come. To the best of our knowledge, the psychometric properties of the 5L in comparison with 3L has never been assessed for multiple cancers simultaneously with value sets of both versions elicited from the country’s general population. A study recently addressed the psychometric properties of the Spanish versions of EQ-5D-Y-3L and EQ-5D-Y-5L in children with cancer. Overall, the study showed that the properties of EQ-5D-Y-5L were better than those of EQ-5D-Y-3L (12). In Iran, the value sets of the 3L in 2017 and 5L in 2022 were derived from the general population (13, 14). The aim of the present study was to assess the psychometric properties of the 5L in Iranian cancer patients and then compare its properties with those of the 3L in the same set of patients.

## Methods

### Study design and data collection

A convenient sample of 650 cancer inpatients and outpatients were selected consecutively from surgery, chemotherapy, and radiotherapy wards in three of the largest governmental cancer centers in three Iranian provinces (Tehran, Esfahan, and Fars) between June 2021 and January 2022. These provinces contain almost than a third of the Iran’s population, and their cancer centers admitted patients from all over the country. Patients with a pathological confirmation of the diagnosis of cancer, healthy cognitive status, and who completed informed consent were recruited in accordance with the ethical standards of the national research committee (approval no. IR. IUMS.1400.394).

Data were gathered using the 3L, 5L, European Organization for Research and Treatment of Cancer Quality of Life Questionnaire (QLQ-C30), and a number of questions on demographic characteristics through face-to-face interviews during a meeting between patients and researchers in patient rooms. Some clinical data were also extracted from the medical records of patients (cancer diagnosis date and service type). The questionnaires were completed in a random sequence to avoid potential bias from an order effect.

### EQ-5D instrument

The EQ-5D is commonly used in two versions: the 3L and 5L. In 2013, the 3L value set was derived from the general public in Iran using the time trade-off method, with a scale ranging from -0.113 (‘33333’ representing the worst health state) to 1 (‘11111’ representing the best health state) (13). In 2022, the Iranian value set of the 5L was generated from the general public using the EuroQoL Group’s Valuation Technology (EQ-VT) protocol, and its scoring function was based on composite time trade-off (cTTO) and discrete choice experiment (DCE) methods. The cTTO was conducted on 86 health states and 196 health states were selected for the DCE valuation. The scores of 5L index range from -1.19 (‘55555’ representing the worst health state) to 1 (‘11111’ representing the best health state) (14).

The QLQ-C30 is a self-reported 30-item questionnaire assessing the HRQoL of cancer patients (15). It contains a global health scale, five functional scales (physical, role, emotional, cognitive, and social), three symptom scales (fatigue, nausea/vomiting, pain), and six single items. All QLQ-C30 items have 4 response levels, except for the two items related to global health status that have 7 response levels. The scoring procedure of QLQC30 scales performs in the two stages. First, the average score of items is calculated in order that computing the raw score. Second, the raw score is converted to a score range from 0 to 100. High score for both functional scales and global health status represents high level of functioning and HRQoL, and higher score of the symptom scales/items represents more health problems (15). The QLQ-C30 version 3 was translated and validated in a sample of cancer patients in Iran (16).

## Data analysis

### Ceiling effect

Ceiling effect for both 5L and 3L was calculated as the proportion of patients reporting ‘no problem’ (level 1) on each dimension and the proportion of ‘no problem’ on all dimensions (health state “11111”). The percentage of ‘no problem’ less than 15% for all dimensions is considered an acceptable percentage of ceiling effect (17). Based on the results of most studies, we hypothesized that the ceiling effect of 5L is lower than 3L (8, 9, 11, 18–22). Since the ceiling effect was very small in some patients, in addition to absolute reduction, a relative reduction was calculated when going from the 3L to 5L using the following formula: (ceiling3L - ceiling5L)/ceiling3L.The differences between ceiling effects of the 3L and 5L were assessed by the McNemar test.

### Inconsistency properties

Inconsistency properties were assessed based on the criteria presented by Janssen et al. (23). An inconsistent response is considered to be present if a response in the 3L was followed by a response in the 5L that was at least two levels away. To quantify inconsistency size, the 3L responses were first recoded on the 5L scale (the 3*L*_5*L*_ as follows: 1 = 1, 2 = 3, 3 = 5, and then calculated as |3*L*_5*L*_ - 5L| - 1; a value of two or more indicated inconsistency.

### Discriminatory power

Discriminatory power of instruments is assessed using informativity’s indices (Shannon index and Shannon Evenness index) (24). The Shannon index (H′) was calculated as follows:


$$H^{\prime }=\sum_{i=1}^{L}p_{i}Log_{2}p_{i}$$

here *H′* denotes the absolute amount of informativity captured, L is the number of levels in a dimension of the EQ-5D, and p_i_ = n_i_/N, the proportion of observations in the i^th^ level (i = 1,…, L), where n_i_ is the observed number of responses in category i and N is the total sample size. The higher the H′, the more the absolute information is. The optimal amount of the H′ is when the responses are evenly distributed across all levels, and was defined as follows: H′max = log_2_L. The Shannon Evenness index (J′) reflects the relative informativity of the questionnaire, regardless of the number of levels, and is calculated by the following formula: J′ = H′/H′max. Higher J′ indicates greater relative information.

### Convergent validity

The convergent validity was assessed by considering the correlation between the EQ-5D dimensions and QLQ-C30 selected scales using the Spearman’s rank correlation coefficient. It is expected that the degree of correlation of each dimension in both EQ-5D with the scales of QLQ-C30 that are theoretically very similar would be higher than those that are theoretically dissimilar (e.g., usual activity of the EQ-5D is expected to be more correlated with physical function than with social function of the QLQ-C30). It is also expected that the correlation between the 3L and QLQ-C30 would be similar to or less than that of the 5L and the other one.

### Known-groups validity

The known-groups validity of both EQ-5D was assessed by testing the difference between the mean score of EQ-5D index in each of the patients’ characteristics using independent sample t-test or ANOVA test. It is expected that the mean EQ-5D index score will be higher for patients with younger age, higher education, and for those receiving less severe treatment strategies and having shorter duration of diagnosis and no comorbidities. The relative precision of both versions was assessed using the relative efficiency (RE). The RE is calculated as the ratio of ANOVA F-statistics (F-statistic5L/F-statistic3L). A RE greater than 1 reveals that the 5L has discriminatory ability greater than the 3L and vice versa (10).

### Reliability

The reliability of both EQ-5D versions and each of the EQ-5D dimensions were assessed respectively using the intra-class correlation (ICC) and Cohen’s weighted kappa coefficients. A sub-sample of 70 patients in a time interval of 1–2 weeks from the first survey was selected and only patients whose response on the general health status (QoL question) remained unchanged were included in final analysis (i.e., to avoid an external shock on data). The ICC was computed using one-way random and single measure methods. Both ICC and kappa were interpreted as follows: “poor,”< 0.40; “fair to good,” 0.40–0.75; and “excellent,” > 0.75 (25).

### Agreement

The Bland–Altman plot is one of the adequate methods for assessing agreement and systematic bias in both measures (26). The plot shows the average value of the 3L and 5L index scores (x-axis) against their difference (y-axis). The 95% limits of agreement were computed as mean difference between the 3L and 5L index scores ±1.96 SD of the differences. The closer to zero the mean difference between scores of both EQ-5D, the better the level of agreement.

All statistical analyses were performed with STATA version 14.0.

## Results

Patients’ demographic and clinical characteristics in both surveys are presented in **Table 1**. In the first survey, of 650 patients who completed the questionnaires, 6 patients were excluded from final analysis because of missing data on the QLQ-C30 scales. The mean age of the patients was 51.71 (SD ± 13.5). The second survey showed that the response of 68 patients on the general health status (QoL question) remained unchanged. The demographic and clinical characteristics of patients in both surveys were very similar, except for gender. The mean age of the patients in the second survey was 52.4 (SD ± 14.3). The largest number of cancer patients was colorectal cancer (22.14%) (**Table 1**)

**Table 1:** Patients’ characteristics in the surveys.

Characteristics	First survey (n = 646) n (%)	Resurvey (n = 68) n (%)
**Gender**		
Male	308 (47.68)	37 (54.41)
Female	338 (52.32)	31 (45.59)
**Age group (years)**		
≤39	133 (20.59)	10 (14.71)
40-49	150 (23.22)	24 (35.29)
50-59	170 (26.32)	14 (20.59)
60-69	130 (20.12)	8 (11.76)
70≤	63 (9.75)	12 (17.65)
**Education**		
Illiterate^a^	128 (19.81)	15 (22.06)
Primary	122 (18.89)	9 (13.24)
Secondary	248 (39.39)	28 (41.18)
University degree	148 (22.91)	16 (23.53)
**Marital status**		
Single	40 (6.19)	5 (7.35)
Divorced or widow	37 (5.73)	4 (5.88)
Married	569 (88.08)	59 (86.77)
**Type of cancer**		
Colorectal	148 (22.14)	26 (38.24)
Lung	132 (20.43)	13 (19.12)
Breast	103 (15.94)	7 (10.29)
Stomach	81 (12.54)	4 (5.88)
Others	187 (28.96)	18 (26.47)
**Current treatment status**		
Chemotherapy	227 (35.19)	11 (16.18)
Radiotherapy	51 (7.19)	4 (5.88)
Chemotherapy and surgery	130 (20.16)	11 (16.18)
Chemotherapy and radiotherapy	75 (11.63)	7 (10.29)
Chemotherapy, radiotherapy, and surgery	162 (25.12)	35 (51.47)
**Duration of disease since diagnosis (months)**		
≤5	276 (42.72)	32 (47.06)
6-11	182 (28.17)	23 (33.82)
12-23	86 (13.31)	7 (10.29)
24≥	102 (15.79)	6 (8.82)
**Comorbidities**		
No	239 (37.00)	28 (41.18)
Yes	407 (63.00)	40 (58.82)

a All patients were able to read or write.

### Ceiling effect

The highest proportion of ‘‘no problems’’ was in the ‘‘self-care’’ dimension for the 3L (68.73) and 5L (64.86), while the lowest proportion was in the ‘‘pain/discomfort’’ (29.26 and 24.61, respectively). Ceiling effects in all dimensions of the 5L were less than those reported for the 3L, and the difference of ceiling effects between the two versions were statistically significant (P< 0.001). Furthermore, the proportion of patients reporting the health state ‘11111’ decreased significantly from 12.07% in the 3L to 9.44% in the 5L (P< 0.001) (**Table 2**).

**Table 2:** Proportion of ‘‘no problems’’ and ceiling effects difference between 3L and 5L.

Dimensions	No problems; n (%)				Ceiling effect difference (%)	
	3L	5L	Chi2	P value^*^	Absolute	Relative
Mobility	380 (58.82)	335 (51.86)	35.27	< 0.001	-6.96	-11.83
Self-care	444 (68.73)	419 (64.86)	12.25	< 0.001	-3.87	-5.63
Usual activities	261 (40.40)	221 (34.21)	33.33	< 0.001	-6.19	-15.32
Pain/discomfort	189 (29.26)	159 (24.61)	17.31	< 0.001	-4.65	-15.89
Anxiety/depression	229 (35.45)	191 (29.57)	27.77	< 0.001	-5.88	-16.58
Full health	78 (12.07)	61 (9.44)	15.21	< 0.001	-2.63	-21.78

*McNemar test.

### Inconsistency

The inconsistent response pairs for each dimension of two versions of the EQ-5D are presented in **Table 3**. The highest proportion of inconsistency was found in the ‘‘anxiety/depression’’ (2.32%), followed by “mobility” (2.16%) (**Table 3**). **Table 3** also shows that the overall proportion and average size of inconsistency were 1.45%, and 1, respectively.

**Table 3:** Inconsistency properties of the 3L and 5L.

Dimensions	Inconsistent response pairs			Inconsistency	
	3L	5L	n (%)	n (%)	Average size
Mobility	1	3	10 (1.55)		
	2	1	1 (0.15)		
		5	3 (0.46)	14 (2.16)	1
Self-care	2	1	3 (0.46)	3 (0.46)	1
Usual activities	2	1	4 (0.62)		
	3	3	2 (0.31)	6 (0.92)	1
Pain/discomfort	1	3	3 (0.46)		
	2	1	1 (0.15)		
		5	5 (0.77)	9 (1.39)	1
Anxiety/depression	1	3	3 (0.46)		
	2	1	6 (0.93)		
		5	1 (0.15)		
	3	3	5 (0.77)	15 (2.32)	1
**Overall**				47 (1.45)	1

### Discriminatory power

The values of absolute (H ′) and relative (J′) informativity were higher for the 5L compared to the 3L, while the absolute informativity in the overall classification system increased from 0.94 for the 3L to 1.38 for the 5L, and relative informativity increased from 0.48 to 0.59. The “usual activities” dimension demonstrated the highest increase in absolute (142.65%) and relative (486.07%) informativity (**Table 4**).

**Table 4:** Shannon index (H′) and Shannon Evenness index (J′) for the 3L and 5L.

Dimension	EQ-5D-3L		EQ-5D-5L		% change from 3L to 5L	
	*H′*	J′	*H′*	J′	*H′*	J′
Mobility	0.86	0.37	1.29	0.82	49.48	119.83
Self-care	0.74	0.32	1.03	0.65	38.71	104.03
Usual activities	1.01	0.43	1.46	0.92	142.65	486.07
Pain/discomfort	0.98	0.42	1.43	0.90	45.58	113.91
Anxiety/depression	0.90	0.38	1.40	0.88	55.82	128.86
Overall	0.94	0.48	1.38	0.59	45.93	22.92

### Convergent validity

The spearman’s rank correlation coefficients showed that the subscales of QLQ-C30 were significantly correlated with the 5L dimensions from 0.37 to 0.60 and with the 3L dimensions from 0.29 to 0.57. The correlation between “anxiety/depression” and “emotional functioning” had the highest value for both the 3L (0.57) and 5L (0.60) (**Table 5**). For each EQ-5D version, the spearman coefficient showed that the degree of correlation between dimensions conceptually relevant was higher than dimensions conceptually irrelevant. As the highest degree of correlation was between the usual activities of EQ-5D and the physical functioning of QLQ-C30 followed by between the pain/discomfort of EQ-5D and the pain of QLQ-C30 (**Table 5**).

**Table 5:** Convergent validity of the 3L and 5L with QLQ-C30 scales.

Dimensions	QLQ-C30 scales					
	Physicalfunctioning	Rolefunctioning	Emotionalfunctioning	Cognitivefunctioning	Socialfunctioning	Pain
**Mobility**						
EQ-5D-3L	0.53	0.46	0.40	0.38	0.38	0.37
EQ-5D-5L	0.58	0.48	0.40	0.42	0.47	0.38
**Self-care**						
EQ-5D-3L	0.52	0.45	0.38	0.37	0.28	0.44
EQ-5D-5L	0.58	0.48	0.39	0.38	0.37	0.45
**Usual activities**						
EQ-5D-3L	0.56	0.53	0.38	0.37	0.36	0.41
EQ-5D-5L	0.59	0.54	0.42	0.42	0.44	0.41
**Pain/discomfort**						
EQ-5D-3L	0.38	0.32	0.41	0.33	0.29	0.53
EQ-5D-5L	0.43	0.38	0.53	0.37	0.37	0.59
**Anxiety/depression**						
EQ-5D-3L	0.36	0.29	0.57	0.36	0.34	0.32
EQ-5D-5L	0.39	0.34	0.60	0.39	0.45	0.37

Spearman’s correlation coefficients. All correlation P-values are less than 0.05.

### Known-groups validity

**Table 6** shows that mean scores of both EQ-5D versions were higher for patients who were male, younger, better educated, married, those receiving less severe current treatment strategies, those having shorter duration of disease since diagnosis, and those without comorbidities. Results also showed that the difference between mean scores of the 3L were significant within age, education, marital status, and treatment status (P<0.05). These differences also were significant within age, education, and treatment status (P<0.05) for the 5L. Furthermore, the RE was more than 1 for education (1.18), treatment status (1.02), duration of disease since diagnosis (1.14), and comorbidities (2.29).

**Table 6:** Known-groups validity and relative efficiency of the 3L and 5L.

Characteristics	n (%)	EQ-5D-3L			EQ-5D-5L			RE
Gender		Mean (SD)	*T or F*	*p* value		*T or F*	*p* value
Male	308 (47.68)	0.639 (0.229)	1.82	0.177^b^	0.424 (0.458)	0.632	0.497^b^	0.34
Female	338 (52.32)	0.663 (0.228)			0.453 (0.483)			
**Age group, years**								
≤39	133 (20.59)	0.709 (0.241)	5.27	0.0003^c^	0.499 (0.487)	2.21	0.066^c^	0.419
40-49	150 (23.22)	0.651 (0.240)			0.436 (0.509)			
50-59	170 (26.32)	0.651 (0.210)			0.439 (0.443)			
60-69	130 (20.12)	0.643 (0.212)			0.457 (0.450)			
70≤	63 (9.75)	0.551 (0.233)			0.288 (0.443)			
**Education**								
Illiterate ^a^	128 (19.81)	0.596 (0.219)	4.04		0.369 (0.441)	4.80	0.072	1.188
Primary	122 (18.89)	0.628 (0.223)	0.005		0.403 (0.479)			
Secondary	248 (39.39)	0.649 (0.210)			0.438 (0.449)			
University degree	148 (22.91)	0.683 (0.256)			0.567 (0.519)			
**Marital status**								
Single	37 (5.73)	0.761 (0.174)	4.91		0.406 (0.380)	2.42	0.089	0.492
Married	569 (88.08)	0.642 (0.237)	0.007		0.430 (0.458)			
Divorced or widow	40 (6.19)	0.644 (0.230)			0.596 (0.477)			
**Current treatment status**								
Chemotherapy	227 (35.19)	0.664 (0.217)	13.38	<0.001	0.380 (0.461)	13.71	<0.001	1.024
Radiotherapy	51 (7.19)	0.834 (0.205)			0.810 (0.327)			
Chemotherapy and surgery	130 (20.16)	0.661 (0.219)			0.390 (0.490)			
Chemotherapy and radiotherapy	75 (11.63)	0.564 (0.268)			0.271 (0.569)			
Chemotherapy, radiotherapy, and surgery	162 (25.12)	0.608 (0.207)			0.520 (0.382)			
**Duration of disease since diagnosis, months**								
≤5	276 (42.72)	0.672 (0.239)	1.42	0.235	0.484 (0.467)	1.63	0.1802	1.147
6-11	182 (28.17)	0.636 (0.223)			0.420 (0.468)			
12-23	86 (13.31)	0.649 (0.177)			0.407 (0.379)			
24≥	102 (15.79)	0.627 (0.248)			0.380 (0.548)			
**Comorbidities**								
No	239 (37.00)	0.672 (0.228)	3.04	0.0817	0.376 (0.487)	6.97	0.008	2.292
Yes	407 (63.00)	0.639 (0.229)			0.477 (0.459)			

^a^All patients were able to read and write.^b^Statistical significance of differences calculated using the t-test.^c^Statistical significance of differences calculated using the ANOVA.

### Reliability

The “mobility” dimension showed the highest coefficient of Kappa for 3L (0.92) and 5L (0.79), while the ‘‘anxiety/depression’’ dimension demonstrated the lowest for both versions (0.66 and 0.64, respectively). The agreement rate ranged from 0.76 (anxiety/depression) to 0.90 (mobility) for the 3L and from 0.73 to 0.86 for the 5L. The ICCs for the 3L and 5L indexes were 0.88 and 0.85, respectively, indicating good reproducibility for both versions (**Table 7**).

**Table 7:** Test–retest reliability of the 3L and 5L.

Dimension	Kappa (95% CI)		Agreement rate (95% CI)	
	3L	5L	3L	5L
Mobility	0.92 (0.90- 1.00)	0.79 (0.67-0.91)	0.90 (0.88-.99)	0.86 (0.78-0.95)
Self-care	0.79 (0.64-0.94)	0.78 (0.65-0.92)	0.89 (0.82- 0.97)	0.86 (0.78-0.95)
Usual activities	0.75 (0.60- 0.91)	0.66 (0.52-0.80)	0.86 (0.78-0.95)	0.75 (0.64-0.85)
Pain/discomfort	0.77 (0.63-0.91)	0.71 (0.58-0.85)	0.87 (0.78-0.95)	0.79 (0.71- 0.89)
Anxiety/depression	0.66 (0.51-0.80)	0.64 (0.44-0.83)	0.76 (0.66-0.86)	0.73 (0.62-0.84)
**Intraclass correlation coefficients (95% CI)**				
**EQ-5D index**	0.88	0.85		

### Agreement

**Figure 1:**
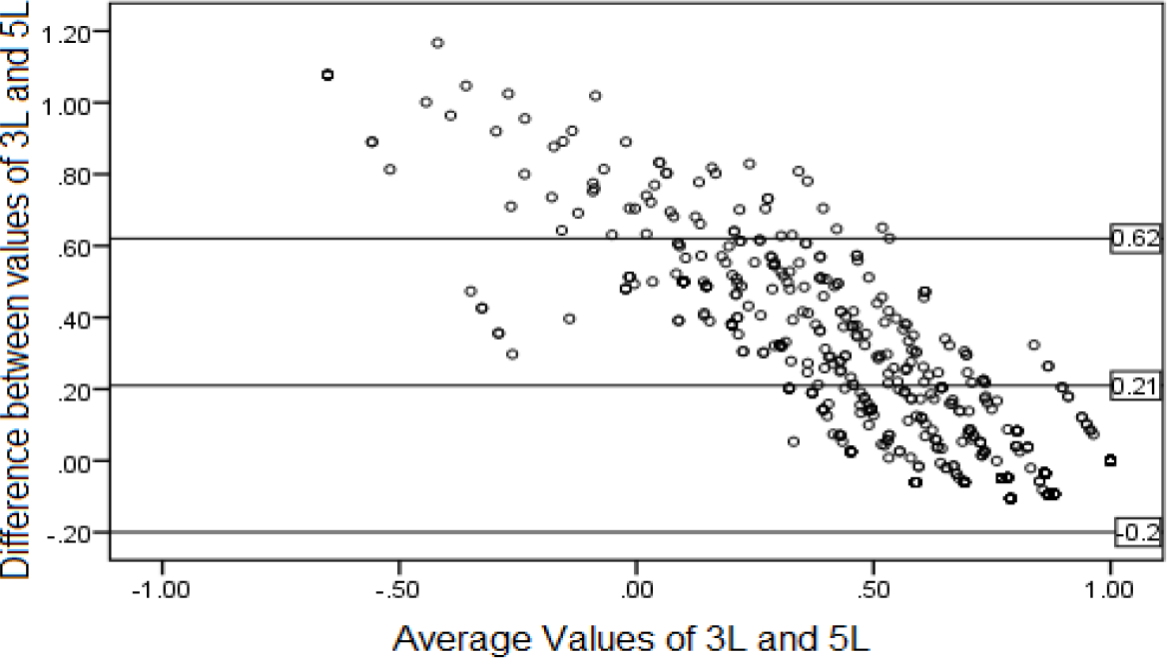
Bland–Altman plot of the 3L and 5L index values.

The Bland-Altman plot showed that mean difference between two versions of the EQ-5D was 0.21. The plot also revealed that 92.57% of the differences of observations between the 3L and 5L were within the 95% limits of agreement (-0.20 to 0.62). 7.43% of the differences distributed above the upper 95% limit, while none of them was below the limit. Differences between scores of the two instruments tended to increase at lower mean values (**Figure 1**).

## Discussion

This is the first study to use national value sets of both EQ-5D versions to assess psychometric properties of 3L and 5L in the context of multiple cancers in terms of ceiling effects, inconsistency informativity, convergent and known-groups validity, reliability, and agreement. The overall mean 5L index score (0.44) was found to be substantially lower than that of the 3L (0.65).

Ceiling effects were observed for both 3L and 5L (12.07 and 9.44, respectively), but were lower than the acceptable limit of 15% reported for instruments (17). These results were also slightly lower than what reported in another study conducted for multiple cancers in Korea (16.8% and 9.7%, respectively). This difference may be due to the lower proportion of more severe cancers in Korea compared to this study. The largest proportion of cancer in Korea (21) was breast (32.9%), followed by colorectal (13.7%), while it was colorectal (22.1%) and lung (20.4%) in this study. Similar to previous studies (11, 21, 27–29), expanding two more levels to the 3L significantly reduced the overall ceiling effect; in this study by 9.44 percentage points, with a relative reduction of 21.79%. The highest proportion of ‘‘no problems’’ responses were reported in “self-care” dimension for both versions. Similar results were found for other diseases such as psoriasis (11), and diabetes (27).

The overall proportion and average value of inconsistent responses were very low (1.45% and 1, respectively) and fell within what was reported in previous studies (0-10.6%), while the highest proportion was in “anxiety/depression” (2.32%) and the lowest in “self-care” (0.46%). The higher variability in the ‘‘anxiety/depression’’ dimension can be explained by its potentially more subjective nature. This was consistent with the finding obtained from studies conducted on general population and diseases (7, 11), while inconsistent with what was reported in Korea (21). The difference can be described due to differences in the proportion of cancer types in the two studies.

The overall discriminatory power increased in all dimensions and in overall classification system when moving from 3L to 5L. The absolute informativity (H ′) of the 5L compared to 3L was as high as 0.44 (45.93%). We also found that relative informativity (J ′) for the overall classification system increased by 22.92%. Similar results were reported in another study conducted on multiple cancers (21), which shows that adding extra levels of severity to the EQ-5D’s descriptive system was used efficiently. Our results of relative informativity were inconsistent with the findings from psoriatic patients and general population (11, 30). It may be due to the differences in the sample selected in our study compared to other studies; cancer patients having more moderate/extreme conditions.

As expected, the correlation coefficients between the 5L dimensions and QLQ-C30 subscales (from 0.37 to 0.60) were higher than those for the 3L dimensions and QLQ-C30 subscales (from 0.29 to 0.57). The pattern of correlation of the 3L and 5L with QLQ-C30 scales was alike, the highest correlation being between “anxiety/depression” and “emotional functioning”, followed by “usual activities” and “physical functioning”, and the lowest was for “self-care” and “social functioning”. This shows that the degree of correlation between the dimensions of both EQ-5D versions that are theoretically very similar to scales of QLQ-C30 was higher than those of the EQ-5D dimensions and QLQ-C30 that are theoretically dissimilar. Therefore, the convergent validity of both EQ-5D versions was confirmed in this study, and supports the results of the study conducted in Korea (21) and other studies (10, 11). Furthermore, the 5L showed stronger correlations with other measures compared to 3L for all dimensions in our study and previous studies (10, 11, 31, 32).

The results of known-groups validity confirmed sufficient ability of both EQ-5D versions to discriminate the difference between their mean scores within demographic and clinical characteristics. The higher mean scores of EQ-5D were associated with higher education, being younger, married, and for those patients who were under less severe treatment strategies, with shorter duration of disease since diagnosis, and no comorbidities. These findings were consistent with previous studies (30, 33, 34), while the result of known-groups for gender was not. Known-groups revealed that female gender had higher utility scores than male for both EQ-5D versions. Higher score for women can be linked to higher number of less severe cancer (breast cancer) among them. However, previous studies revealed that the mean score of EQ-5D in breast cancer was higher than other cancers such as digestive system cancer (35). Furthermore, the RE results demonstrated a higher discriminatory efficiency for the 5L for education variable and all clinical variables. The higher discriminatory efficiency of the 5L in clinical variables was reported in studies conducted on psoriatic (11) and acute myeloid leukemia (36) patients.

ICC and Kappa coefficients confirmed a high reproducibility for both EQ-5D versions in our study. As the ICC values for the 3L (0.88) and 5L (0.83) indicated an excellent level of 0.75 reproducibility (25), Kappa also revealed that all dimensions of both EQ-5D fell within the range reported for a good level of reproducibility (25). Reliability was better than what reported in Korea (21). This can be explained by the fact that the average time interval between the two surveys in our study was shorter (7.5 versus 11.5 days), therefore the condition of the patients might have changed less. Similar to the study in Korea, the results of our reliability showed that the 5L was less reproducible than the 3L in all dimensions. This may be due to a large number of levels in the 5L that may affect recall bias. This result was not supported in studies conducted in the general public (37) and in patients with diabetes (27). This difference can be explained by the differences in the health status of target populations.

The Bland–Altman plot revealed that the proportion of 92.57% of the differences of observations between the 3L and 5L was within the 95% limit of agreement (-0.20 to 0.62). Although the high proportion of observations fell within the limit, these differences of observations were not distributed symmetrically. The plot showed that the agreement between the two instruments was weaker for patients in more severe health states (i.e., patients with lower utility value) where the majority of the differences in utility scores lied outside the limits of agreement. That is, the 3L overestimated the utilities for more severe health states and underestimated them for better health states. This might be due to differences between floor effects in 3L and 5L (-0.113 and -1.19, respectively), and to the presence of more negative values in the Iranian value set of the 5L compared to the 3L value set. One limitation that should be noted is that we did not assess responsiveness in the study, which is an important measurement property.

## Conclusion

Findings suggest that the 5L was better than the 3L in terms of ceiling effect, inconsistency, discriminatory power, convergent validity, and relative precision. Both versions of the EQ-5D demonstrated good known-groups validity and reliability as well as 92.57% of the differences of observations between the 3L and 5L was within the 95% limit of agreement. It is thus recommended to use the 5L in cancer research or clinical practice.

## Data availability statement

The raw data supporting the conclusions of this article will be made available by the authors, without undue reservation. Requests to access these datasets should be directed to Hamery7@yahoo.com.

## Ethics statement

The studies involving human participants were reviewed and approved by the Iranian national research committee (approval no. IR. IUMS.1400.394). The patients/participants provided their written informed consent to participate in this study.

## Author contributions

Study design and statistical analysis and interpretation of thedata: HA and NM. Drafting of the manuscript: HA and TGP andHS. Critical revision of the manuscript for important intellectualcontent: HA, TGP and MM. All authors contributed to the article and approved the submitted version.
